# Provision of immediate postpartum contraception to women living with HIV in the Eastern Cape, South Africa; a cross-sectional analysis

**DOI:** 10.1186/s12978-020-01049-9

**Published:** 2020-12-09

**Authors:** Oladele Vincent Adeniyi, Anthony Idowu Ajayi, Oluwaseyi Dolapo Somefun, John Shearer Lambert

**Affiliations:** 1 Department of Family Medicine & Rural Health, Faculty of Health Sciences, Walter Sisulu University, Mthatha/East London Hospital Complex, Cecilia Makiwane Hospital, East London, South Africa; 2 Population Dynamics and Sexual and Reproductive Health, African Population and Health Research Centre, APHRC Campus, Manga Close, Nairobi, Kenya; 3 Demography and Population Studies (DPS), University of the Witwatersrand, Johannesburg, South Africa; 4 Department of Infectious Diseases, Medicine and Sexual Health. Mater, Rotunda and University College, Dublin, Ireland

**Keywords:** Eastern Cape, Immediate postpartum contraception, Parturient women with HIV, Long-acting reversible contraception, Permanent contraception, Short-acting contraception, South Africa

## Abstract

**Background:**

Universal access to contraception is an important strategy adopted by the South African government to reduce the high rate of unintended pregnancies, especially in women living with HIV. In this article, we describe the choices of contraception and also, examine the influencing factors of the choices of contraception in the immediate postpartum period in parturient women with HIV in the Eastern Cape, South Africa.

**Methods:**

In this prospective cross-sectional study, 1617 parturient women with HIV completed a survey on the choice of contraception received in the immediate postpartum period (within 72 h) across three large maternity services in the Eastern Cape between September 2015 to May 2016. Additional information was extracted from their medical records. Choices of contraception were categorised as; short-acting (injectables), long-acting reversible (intrauterine device and implants) and permanent contraception (tubal ligation). Adjusted and unadjusted logistic regression models were employed to determine the influencing factors of the choices of contraception received by the cohort.

**Results:**

Participants were predominantly single (69.1%), unemployed (75.1%), had a grade 7–12 level of education (88.4%) and were HIV positive before their index pregnancy (81.3%). The prevalence of immediate postpartum contraception was high (n = 1507; 93.2%) with Injectables being the preferred choice in the majority of the participants (n = 1218; 75.3%). After controlling for all relevant covariates, single marital status was associated with a higher likelihood of immediate postpartum contraceptive initiation (AOR; 1.82 95% CI 1.10–3.03). Overall, women were more likely to initiate a long-acting reversible and irreversible methods when older than 35 years and having had more than two children.

**Conclusions:**

We found a high prevalence of immediate postpartum contraception with a preference for Injectables in the study setting. Long-term monitoring of this cohort will elucidate on contraceptive discontinuation and risk of unintended pregnancies in the region.

**Plain English Summary:**

Ensuring universal access to contraceptives is an important strategy to reduce the rate of unintended pregnancies at the population level. This strategy was adopted by the South African government with a vision of stemming the tide of unintended pregnancies among women living with HIV. In this study, the choices of contraception adopted by women living with HIV following the delivery of their babies were explored. In addition, the study highlights the factors that predict these choices. Participants were asked the choice of contraception they had received prior to being discharged from the maternity centres where they had delivered their babies. The various types of contraception were then categorised by their duration of action. Three distinct groups emerged; short-acting injectables, long acting reversible contraceptives and permanent methods. Of the 1617 women included in the study, 1117 were single and 1314 knew their HIV status prior to the onset of the index pregnancy. Almost all the women (1507 out of 1617) received one form of contraception before leaving the hospital. Many women (1218 out of 1617) chose injectable contraception (short-acting contraception) over the other types of contraception. Women who were older than 34 years and who had three or more children were more likely to choose a long-acting reversible contraceptive and permanent method over the short-acting contraception or nothing. In conclusion, given the short duration of action of the predominant method adopted by these women, a long-term follow up of the study participants will provide more information on the continued use of contraception and risk for unintended pregnancies.

## Background

Unintended pregnancy remains a serious public health problem, especially within the first 12 months of previous delivery [1]. Unintended pregnancy often results in abortion, and when it occurs shortly after childbirth it could result in preterm labour, intra-uterine growth restriction and stillbirth [2]. In addition, unintended pregnancies are associated with increased levels of stress, decreased quality of life, late initiation of antenatal care, and maternal deaths [3–6]. Given its deleterious consequences, prevention of unintended pregnancy is a critical component of all global development goals, including the Sustainable Development Goals. Implementation of an effective contraception in the immediate postpartum period is a proven intervention to mitigate some of these adverse outcomes [7]. Interestingly, parturient women are largely accessible in the immediate postpartum period for important reproductive health interventions which could shape the future of their health and that of their wards [8]. According to the World Health Organisation (WHO), implementation of family planning (FP) should be an integral component of the existing maternal and child health services [9]. This strategy was adopted by the South African government as one of the multi-pronged prevention approaches toward eliminating vertical transmission of HIV [10].

About 36.9 million people are currently living with HIV/AIDS globally, 70% of them live in sub-Saharan Africa (SSA) [11, 12], and the majority of them are females in their reproductive age [13]. Approximately 1.5 million pregnancies occurred in women living with HIV in developing countries [14], and most of these pregnancies are unplanned or unintended [15–17]. From an economic perspective, supporting women living with HIV (WLWH) to use contraception is a cost-effective approach for preventing MTCT of HIV [18, 19]. Even though substantial progress has been recorded in the prevention of mother-to-child transmission of HIV (PMTCT), a recent UNICEF report indicated that about 180,000 new HIV infections occurred among children [20]. Perinatal transmission of HIV is highest in sub-Saharan Africa [21] and could be reduced by addressing the high rate of unintended pregnancies among women living with HIV.

South Africa has a heavy burden of HIV; 7,7 million people living with HIV (a prevalence of 20.4% among adults 15–49 years) [13, 22], and an estimated 12,000 children were newly infected with HIV due to MTCT in 2016 [11]. The majority of pregnancies among women living with HIV in the country are unintended; ranging from 62% in Johannesburg, Gauteng province to 71% in Buffalo/Amathole districts of Eastern Cape province [15, 16] suggesting that prevention of unplanned and unwanted pregnancies could contribute to reducing MTCT of HIV. Family planning has been integrated into PMTCT programmes at all the primary health care centres in South Africa with the goal of reducing unplanned pregnancies, ensure optimal spacing of births and ultimately reduce MTCT of HIV. Services integration provides an opportunity for consolidating care for patients across the country. In practice, women accessing care for HIV are counselled about contraception and offered for free in all public health facilities across the country. Also, women are counselled during ante-natal care, labour and immediate postpartum period about making choices of contraception. A good measure of the impact of ante-natal counselling and PMTCT service’ integration is to assess the uptake of contraception in the immediate postpartum period and long-term follow-up of the cohort of parturient women.

While there are studies that have examined contraceptive uptake among women with HIV [23–26], we did not come across any studies focusing on immediate postpartum contraceptive use in South Africa, a country with a high HIV prevalence. As such, the extent of the implementation of the policy on integration of family planning and PMTCT services at the health facilities and various districts in the country remains uncertain. This study describes the prevalence and choices of immediate postpartum contraception among parturient women living with HIV in the Eastern Cape, South Africa. In addition, the study further examines the influencing factors of the choices of immediate postpartum contraception in the cohort. We know from existing studies that women who gave birth via caesarean section are more likely to initiate long-acting and permanent contraception [23–26]. Our study will contribute to enhancing the understanding of family planning needs of women living with HIV, which is important for improving services and reducing mother to child transmission of HIV in the resource constrained Eastern Cape Province of South Africa.

## Methods

### Study design and setting

We conducted a cross-sectional analysis of the baseline dataset of all women enrolled in the East London Prospective Cohort Study [22]. The East London Prospective Cohort Study aimed at examining the PMTCT outcomes in the Eastern Cape province, following the implementation of the lifelong antiretroviral therapy (ART) for all pregnant women in South Africa. The baseline dataset was created between September 2015 and May 2016. Three large maternity services in the Buffalo/Amathole districts of the Eastern Cape Province were purposively selected for this study. These facilities together serve over 1.6 million people from rural, semi-urban and urban areas of the province and deliver an average of 1150 pregnant women monthly. In addition, these facilities receive referrals from all hospitals and midwife obstetric units (MOUs) across the region.

### Participants recruitment and data collection procedures

The study population comprises all HIV infected women (N = 1709) who gave birth over the study period (September 2015 to May 2016). Participants were included in the study if they were HIV positive and gave birth in the selected maternity facilities during the study period without any exclusion. Participants were recruited consecutively at the postnatal wards of the maternity centres within 24 h of vaginal delivery and 72 h of caesarean section delivery. There was no dissent among the eligible participants throughout the study period. Three research assistants (undergraduate students), who were proficient in the local language (IsiXhosa) were trained for 2 days and allocated per study site. The research assistants completed an interviewer-administered questionnaire, which was pre-piloted specifically for this study. Data were captured using Android tablet with pre-installed questionnaire linked to the electronic database created for this study. This database archived all the dataset of the study in the Paediatric ART (antiretroviral therapy) Data Management Tool hosted on the Eastern Cape Department of Health Server.

Participants were interviewed in private rooms provided by the respective health facilities for this study. Additional information on the clinical characteristics such as viral load, CD4 counts, date of antenatal booking and ART medications were extracted from the maternity records of each participant during the interview. Three research nurses (allocated per site) were recruited to draw blood samples from participants with missing viral load and CD4 counts. The research nurses also provided assistance with the extraction of information from the medical records of the participants.

## Measurement

The main outcome measure was the prevalence of immediate postpartum contraceptive initiation (within 72 h of childbirth). Participants were asked whether they had initiated any type of contraception after delivery of their index pregnancy and if so, to state the choice of contraception received. We validated the participants’ report of contraception received and also, confirmed the specific method and category of contraception received by the participants from their medical records. There were no discrepancies between their medical records and their self-reports. The contraceptive methods reported by the participants include Nuristerate, Depo Provera, Intrauterine device, Implants and Bilateral tubal ligation. To aid our analysis, we classified Nuristerate and Depo Provera as shorting acting reversible contraceptives [27]. Intrauterine device and Implants were grouped together because both are long acting reversible contraceptive methods [28]. Tubal ligation was grouped alone as long acting irreversible method (permanent).

Our review of the previous studies and health behaviour theories [23–26], shows that individual and clinical factors could influence contraceptive use. For example, studies have shown that age, income, education, place of residence, parity, marital status are important predictors of contraceptive use. In this study, we considered the age, parity, abortion history, marital status, alcohol use, mode of child delivery and prenatal HIV status (status at booking) to be relevant independent variables. We measured age as a continuous variable. But we later categorised age into 14–24 years, 25 to 34 years and 35 to 44 years for further analysis. Participants were asked to state whether they were married, single or cohabiting. Also, they were asked to indicate whether they ever consume alcohol, and if they did so during pregnancy or had stopped drinking during pregnancy. While alcohol has no direct effect on the efficacy of hormonal contraceptive, evidence suggests that it negatively influences the behaviour of the women and thus, impacts on the continued use [29]. Also, participants were asked to state the number of children they had ever delivered, including the index baby (parity). The number of children had could significantly influence if they will use contraceptives as well as their method of choice.

Participants were asked to indicate whether they had ever terminated any pregnancy (induced abortion) and number of times they ever terminated pregnancy. Clinical characteristics of participants, such as the timing of initiation of antenatal care, HIV status at first presentation and mode of delivery were extracted from medical records.

## Data analysis

Complete data were imported into the IBM Statistical Package for Social Sciences (Version 25) for Windows (SPSS Inc., Chicago, Illinois, USA). Data were cleaned to get rid of all data entry errors. This was done by first running a simple frequency for all variables. We then examine the results for possible outliers and those attributable to data entry errors were corrected. All variables of interest were subjected to descriptive statistics. Mean and standard deviations were computed for continuous variables. Frequency count, chi-square statistics and binary logistic regression were estimated for categorical variables. We first examined the factors associated with immediate postpartum contraceptive initiation using adjusted and unadjusted logistic regression models. Also, we fitted adjusted and unadjusted logistic regression models to understand the factors associated with initiation of tubal ligation—a long acting irreversible method—, intrauterine device and implants, and injectables. All analyses were performed at 95% confidence level and p-values less than 0.05 were deemed to be statistically significant. The variables included in our multivariable analysis was based on our review of the literature [23–26], on factors associated with immediate postpartum contraception. About 90 participants did not complete the questions on contraception, as such, we performed a sensitivity analysis by comparing the demographic characteristics of participants that did not respond to the question (n = 90) on contraception to those that responded (n = 1617) and found no significant difference.

## Results

A total of 1617 participants who had complete responses to the main outcome measure were included in the final analysis. Most participants were single (69.1%), unemployed (75.1%), had a grade 7–12 level of education (88.4%) and were HIV positive before their index pregnancy (81.3%) (Table 1). Participants’ average age was 29.66 [standard deviation (SD) = 6.18] years.

**Table 1:** Demographical characteristics of respondents

Variable	Frequency (n-1617)	Percentages (%)
Age		
20 and below	111	6.9
21–25	348	21.5
26–30	436	27.0
31–35	415	25.7
36–40	247	15.3
Above 40 years above	60	3.7
Marital status		
Married	299	18.5
Single	1118	69.1
Cohabiting	177	10.9
Divorced/separated	23	1.4
Educational level		
No formal education	5	0.3
Grade 1–6	75	4.7
Grade 7–12	1406	88.4
Tertiary	104	6.5
Employment		
Unemployed	1204	75.1
Employed	399	24.9
Smoking status		
Smoked during pregnancy	86	5.3
Quit smoking during pregnancy	76	4.7
Never smoked	1455	89.7
Alcohol use		
Drank during pregnancy	212	13.1
Stopped drinking during pregnancy	416	25.7
Never drank alcohol	988	61.1
Prenatal HIV status		
Positive	1303	81.3
Negative	82	5.1
Unknown	217	13.5
Abortion history		
Never had abortion	1310	81.0
Ever terminated a pregnancy	307	19.0
Parity		
One child	485	30.0
Two children	582	36.0
Three children	334	40.7
Four children and more	216	13.4

### Prevalence of immediate postpartum contraceptive initiation

The majority of the participants (93%) had initiated one form of contraception within three days’ post-delivery. Short-acting contraception (Nuristerate and Depo-Provera) was the predominant method received by the participants (n = 1218; 75.3%). A small proportion of women initiated the long-acting reversible contraception (implants and IUD) (8.5%) and permanent method (tubal ligation) (8.2%).

### Association between demographic characteristics and immediate postpartum contraceptive initiation

In the chi-square test; age, place of residence, educational status, employment, parity and abortion history were not significantly associated with the uptake of immediate postpartum contraception. Only alcohol use, being married and unknown/negative status at first antenatal care were negatively associated with the uptake of immediate postpartum contraception (Table 2).

**Table 2:** Relationship between demographic characteristics and uptake of immediate postpartum contraception

Variable	Yes	No	*P*-value
Age			
20 and below	98 (88.3)	13 (11.7)	0.365
21–25	324 (93.1)	24 (6.9)	
26–30	410 (94.0)	26 (6.0)	
31–35	387 (93.3)	28 (6.7)	
36–40	233 (94.3)	14 (5.7)	
Above 40 years above	55 (91.7)	5 (8.3)	
Marital status			
Married	273 (91.3)	26 (8.7)	*0.011*
Single	1054 (94.3)	64 (5.7)	
Cohabiting	157 (88.7)	20 (11.3)	
Divorced/separated	23 (100.0)	0 (0.0)	
Area			
Rural	494 (93.7)	33 (6.3)	0.646
Semi-Urban	706 (93.6)	48 (6.4)	
Urban	284 (92.2)	24 (7.8)	
Educational level			
No formal education	4 (80.0)	1 (20.0)	0.628
Grade 1–6	71 (94.7)	4 (5.3)	
Grade 7–12	1310 (93.2)	96 (6.8)	
Tertiary	96 (92.3)	8 (7.7)	
Employment			
Unemployed	1126 (93.5)	78 (6.5)	0.218
Employed	368 (92.2)	31 (7.8)	
Alcohol use			
Drank during pregnancy	193 (91.0)	19 (9.0)	*0.045*
Stopped drinking during pregnancy	380 (91.3)	36 (8.7)	
Never drank alcohol	934 (94.4)	55 (5.6)	
HIV status at first antenatal booking			
Positive	1233 (94.6)	70 (5.4)	*< 0.001*
Negative	69 (84.1)	13 (15.9)	
Unknown	190 (87.6)	27 (12.4)	
Abortion history			
Never had abortion	1217 (92.9)	93 (7.1)	0.199
Ever terminated a pregnancy	290 (94.5)	17 (5.5)	
Parity			
1	448 (92.4)	37 (7.6)	0.216
2 and 3	862 (94.1)	54 (5.9)	
4 and more	197 (91.2)	19 (8.8)	

P-values estimated using Pearson chi-square test

### Multivariable findings

As shown in Table 3, only having a negative HIV status at the first antenatal booking was significantly associated with lower likelihood of immediate postpartum contraceptive initiation in the unadjusted model. After controlling for all relevant covariates, single marital status was associated with a higher likelihood of immediate postpartum contraceptive initiation. While having a negative HIV status at first antenatal booking was associated with a reduced odd of immediate contraceptive initiation.

**Table 3:** Adjusted and unadjusted logistic regression model showing factors associated with immediate postpartum contraceptive initiation among women with HIV

Variables	Unadjusted model	Adjusted model
	UOR (CI)	UOR (CI)
Age		
14–24 years	1	1
25 to 34 years	1.52 (0.96–2.40)	1.32 (0.78–2.25)
35 to 44 years	0.42 (0.73–2.13)	1.16 (0.59–2.27)
Marital status		
Married	1	1
Single	1.60 (1.0–2.58)	1.82 (1.10–3.03)*
Cohabiting	0.75 (0.40–1.38)	0.93 (0.49–1.76)
Educational level		
Tertiary education	1	1
Grade 7–12	1.10 (0.52–2.32)	1.05 (0.48–2.29)
Grade 6 or less	1.20 (0.38–3.82)	1.16 (0.35–3.84)
Employment status		
Employed	1	1
Unemployed	1.22 (0.79–1.87)	1.23 (0.78–1.94)
Place of residence		
Rural	1	1
Semi Urban	1.08 (0.70–1.68)	1.21 (0.77–1.92)
Urban	0.87 (0.51–1.48)	1.12 (0.64–1.96)
Alcohol use		
Drank during pregnancy	1	1
Stopped drinking during pregnancy	1.04 (0.58–1.86)	0.84 (0.46–1.55)
Never drank alcohol	1.67 (0.97–2.88)	1.37 (0.77–2.43)
Abortion of history		
Never terminated a pregnancy	1	1
Ever terminated a pregnancy	1.30 (0.77–2.22)	1.31 (0.75–2.27)
Parity		
One	1	1
Two	1.63 (0.98–2.71)	1.64 (0.95–2.84)
Three	0.98 (0.58–1.65)	0.97 (0.53–1.76)
Four and above (ref)	0.86 (0.48–1.53)	0.83 (0.41–1.67)
Mode of delivery		
Caesarean section delivery	1	1
Vaginal delivery	1.11 (0.75–1.64)	1.09 (0.73–1.62)
HIV status at first antenatal care booking		
Positive	1	1
Negative or unknown	0.37 (0.25–0.56)***	0.40 (0.26–0.62)***

*** The *P-*value < 0.001; * *P-*value < 0.05; ref: reference; UOR: Unadjusted Odds Ratio; CI: Confidence Interval

We presented the results on factors associated with initiation of short acting reversible contraceptive methods in Table 4. In the unadjusted logistic regression analysis, only older age (35–44 years), having three or more children and having ever terminated a pregnancy were associated with reduced odds of initiating short acting reversible contraceptives. While single marital status, having never drank alcohol, and vaginal mode of delivery were associated with higher odds of initiating a short-acting reversible contraceptives.

**Table 4:** Adjusted and unadjusted logistic regression model showing factors associated with the initiation of a short acting reversible contraceptive method

Variables	Unadjusted model	Adjusted model
	UOR (CI)	UOR (CI)
Age		
14–24 years	1	1
25 to 34 years	0.87 (0.64–1.19)	1.25 (0.86–1.82)
35 to 44 years	0.38 (0.27–0.53)***	0.65 (0.42–1.00)
Marital status		
Married	1	1
Single	1.63 (1.22–2.17)*	1.74 (1.03–1.98)*
Cohabiting	0.86 (0.57–1.28)	0.94 (0.60–1.47)
Educational level		
Tertiary education	1	1
Grade 7–12	1.11 (0.70–1.74)	1.13 (0.68–1.88)
Grade 6 or less	0.94 (0.48–1.81)	0.94 (0.44–2.00)
Employment status		
Employed	1	1
Unemployed	1.16 (0.89–1.51)	1.08 (0.80–1.45)
Place of residence		
Rural	1	1
Semi Urban	1.13 (0.87–1.46)	1.19 (0.89–1.60)
Urban	0.92 (0.67–1.27)	1.07 (0.74–1.54)
Alcohol use		
Drank during pregnancy	1	1
Stopped drinking during pregnancy	1.19 (0.82–1.70)	1.13 (0.75–1.70)
Never drank alcohol	1.73 (1.25–2.41)*	1.76 (0.1.21–2.57)*
Abortion of history		
Never terminated a pregnancy	1	1
Ever terminated a pregnancy	0.71 (0.54–0.94)*	0.93 (0.68–1.28)
Parity		
One	1	1
Two	0.97 (0.70–1.34)	0.92 (0.64–1.33)
Three	0.36 (0.26–0.49)***	0.36 (0.24–0.54)***
Four and above (ref)	0.28 (0.19–0.40)***	0.27 (0.17–0.42)***
Mode of delivery		
Caesarean section delivery	1	1
Vaginal delivery	4.48 (3.47–5.79)***	5.32 (4.03–7.04)***
HIV Status at first antenatal care booking		
Positive	1	1
Negative or unknown	0.95 (0.71–1.27)	0.85 (0.61–1.19)

*** The *P-*value < 0.001; * *P-*value < 0.05; ref: reference; UOR: Unadjusted Odds Ratio; CI: Confidence Interval.

After controlling for all relevant covariates, the effect of age on initiation of short-acting reversible contraceptive was no longer statistically significant even though the direction of effect remains. Women who were single and those who never drank alcohol were approximately twice more likely to initiate a short-acting reversible contraceptive method compared to married women and those who drank alcohol during pregnancy. The effect of abortion history was no longer statistically significant. However, women who gave birth through vaginal were over five times more likely to initiate a short-acting reversible contraceptive methods relative to those who gave birth via caesarean section.

The factors associated with initiation of long-acting contraceptives are presented in Table 5. In the unadjusted model, older age (35 to 44 years), having ever terminated a pregnancy, and parity of three or more children were significantly associated with a higher likelihood of initiating a long-acting reversible contraceptive method. While being single, having never drank alcohol, negative or unknown HIV status at first antenatal attendance, and vaginal mode of delivery were significantly associated with lower odds of initiating a long-acting reversible contraceptive method. After adjusting for all relevant variables, older age (35 to 44 years) remained significantly associated with initiation of long-acting reversible contraceptive methods even the effect size reduced by about half. Marital status was no longer significantly associated with initiation of a long-acting reversible contraceptive method. Having ever used alcohol remained significantly associated with lower odds of initiating a long-acting contraceptive method. Women who had given birth to three or more children were four to six times more likely to initiate a long acting reversible contraceptive method compared to those who had only one child. Lastly, negative or unknown HIV status during the first antenatal care booking was associated with lower odds of initiating a long-acting reversible contraceptive method.

**Table 5:** Adjusted and unadjusted logistic regression model showing factors associated with initiation of a long-acting reversible contraceptive methods

Variables	Unadjusted model	Adjusted model
	UOR (CI)	UOR (CI)
Age		
14–24 years	1	1
25 to 34 years	1.62 (1.08–2.41)*	0.92 (0.58–1.48)
35 to 44 years	4.14 (2.73–6.27)***	2.01 (1.19–3.40)*
Marital Status		
Married	1	1
Single	0.66 (0.48–0.92)*	0.82 (0.56–1.19)
Cohabiting	1.05 (0.66–1.65)	1.01 (0.59–1.71)
Educational level		
Tertiary education	1	1
Grade 7–12	0.92 (0.55–1.53)	0.82 (0.56–1.19)
Grade 6 or less	1.17 (0.56–2.45)	1.01 (0.59–1.71)
Employment status		
Employed	1	1
Unemployed	0.90 (0.67–1.21)	0.87 (0.48–1.58)
Place of residence		
Rural	1	1
Semi Urban	0.89 (0.66–1.19)	0.91 (0.64–1.29)
Urban	1.04 (0.72–1.50)	0.98 (0.63–1.51)
Alcohol use		
Drank during pregnancy	1	1
Stopped drinking during pregnancy	0.82 (0.54–1.23)	0.75 (0.46–1.20)
Never drank alcohol	0.62 (0.43–0.91)*	0.52 (0.34–0.82)*
Abortion of history		
Never terminated a pregnancy	1	1
Ever terminated a pregnancy	1.69 (1.25–2.29)*	1.15 (0.80–1.65)
Parity		
One	1	1
Two	1.43 (0.95–2.15)	1.52 (0.97–2.40)
Three	4.06 (2.72–6.05)***	3.91 (2.44–6.29)***
Four and above (ref)	5.08 (3.31–7.81)***	5.55 (3.23–9.51)***
Mode of delivery		
Caesarean section delivery	1	1
Vaginal delivery	0.13 (0.90–0.18)***	0.09 (0.07–0.14)***
HIV status at first antenatal care booking		
Positive	1	1
Negative or unknown	0.55 (0.38–0.82)*	0.60 (0.39–0.93)*

*** The *P-*value < 0.001; * *P-*value < 0.05; 1: reference; UOR: Unadjusted Odds Ratio; CI: Confidence Interval.

We presented the factors associated with choosing tubal ligation in Table 6. In the unadjusted model, women were more likely to choose bilateral tubal ligation if they were over 25 years, had ever terminated a pregnancy, and had two or more children. They were less likely to choose tubal ligation if they were single and diagnose with HIV during their index pregnancy. In the adjusted model only older age, having ever terminated a pregnancy and having two and more children remained statistically significant. Older women (35 years and above) were twelve times more likely to choose bilateral tubal ligation compared to younger women (14 to 24 years). Women who self-reported an induced abortion were twice more likely to choose bilateral tubal ligation compared to those who never induced abortion. Women who had three children and those with four or more children were fifteen and thirty times more likely to choose bilateral tubal ligation compared with those with one child.

**Table 6:** Adjusted and unadjusted logistic regression model showing factors associated with choosing bilateral tubal ligation

Variables	Unadjusted model	Adjusted model
	UOR (CI)	UOR (CI)
Age		
14–24 years	1	1
25 to 34 years	9.35 (2.25–38.84)*	3.31 (0.76–14.30)
35 to 44 years	54.93 (13.41–224.96)***	12.17 (2.81–52.66)***
Marital status		
Married	1	1
Single	0.63 (0.41–0.97)*	1.17 (0.73–1.91)
Cohabiting	1.06 (0.59–1.92)	1.36 (0.70–2.66)
Educational level		
Tertiary education	1	1
Grade 7–12	0.94 (0.46–1.90)	0.62 (0.28–1.37)
Grade 6 or less	1.75 (0.69–4.46)	0.93 (0.311–2.77)
Employment status		
Employed	1	1
Unemployed	0.91 (0.61–1.36)	0.93 (0.31–2.77)
Place of residence		
Rural	1	1
Semi Urban	0.81 (0.54–1.21)	0.65 (0.41–1.01)
Urban	1.03 (0.63–1.68)	0.84 (0.48–1.47)
Alcohol use		
Drank during pregnancy	1	1
Stopped drinking during pregnancy	0.78 (0.45–1.35)	0.96 (0.51–1.80)
Never drank alcohol	0.65 (0.40–1.07)	0.68 (0.38–1.20)
Abortion of history		
Never terminated a pregnancy	1	1
Ever terminated a pregnancy	2.23 (1.52–3.29)***	1.66 (1.08–2.57)*
Parity		
One	1	1
Two	5.72 (1.69–19.19.36)*	3.64 (1.05–12.64)*
Three	30.3 (9.38–97.88)***	15.04 (4.48–50.49)***
Four and above (ref)	56.2 (17.36–182.13)**	20.62 (6.06–70.015)
HIV status at first antenatal care booking		
Positive	1	1
Negative or unknown	0.41 (0.22–0.75)*	0.58 (0.30–1.13)

*** The *P-*value < 0.001; * *P-*value < 0.05; 1: reference; UOR: Unadjusted Odds Ratio; CI: Confidence Interval.

## Discussion

This study determines the prevalence of immediate postpartum contraceptive initiation among women with HIV and describes their choices of contraception in the context of family planning and HIV services’ integration in the Eastern Cape, South Africa. In addition, the study elucidates the influencing factors of the choices of contraception received by parturient women with HIV in the immediate postpartum period. The prevalence of immediate postpartum contraception of 93.2% in this cohort is very commendable. This finding provides evidence of the effectiveness of family planning and HIV services’ integration policy which aims to promote universal access to contraception in every maternity facility in the country.

The high prevalence reported here should be interpreted with caution for two reasons. Given the high rate of unplanned pregnancies in women living with HIV in the region [15, 16], a long-term follow up of this cohort is therefore recommended in order to gain a broader understanding of contraception in women living with HIV in the region. Another plausible explanation could be that a large proportion of women in need of contraception were easily accessible in the maternity in the immediate postpartum period. Perhaps, the decision to receive contraception at the time could have been influenced by subtle peer pressure from other parturient women or coercion by clinicians is unclear in the present study. Future studies should examine the effect of providers’ bias and coercion on the choices of contraception in the region, given South Africa’s history and a body of literature establishing contraceptive coercion [30, 31].

Consistent with previous studies [32–35], our study showed that short-acting methods were the preferred choices of contraception by the parturient women with HIV. It is important to note that a high prevalence of short-acting contraception may not guarantee continuous use beyond the postpartum period. Long-term follow up of this cohort to monitor retention in care and continuity of contraception, including method will enhance our understanding of family planning needs of women in the region. A study previously reported that method convenience and health care providers’ recommendations often influence the choice of contraception of women [17]. It is vital to educate women further on the advantages of long-acting reversible methods and encourage them to use these methods. Long-acting reversible methods are highly effective and would nearly eliminate the chances of unplanned pregnancies, given that they are less prone to human errors.

Our study showed that uptake of contraception differs by marital status; single women (94.3%) compared with married women (91.3%) and women in cohabiting relationship (88.7%). These rates are significantly higher than the current use of contraception reported for the general population of women aged 15–49 years in 2016, which ranged from 51% in Frere State to 65% in KwaZulu Natal province [29]. However, it should be noted that there was no significant difference in the uptake of contraception by marital status after adjusting for other co-variates in the logistic regression model analysis.

Our study also showed that women who were previously diagnosed with HIV before their index pregnancy were more likely to use short-acting, long-acting and permanent contraceptive methods compared to women diagnosed during their index pregnancy. This finding suggests that women with longer duration of infection (diagnosed before the index pregnancy) may have had time to consider their fertility intentions and possibly consider the index pregnancy to be their last pregnancy. Those diagnosed with HIV during the index pregnancy may still be reflecting on their fertility intentions and the impact of HIV infection on future pregnancies.

Our study showed that women who gave birth via caesarean section had a higher likelihood of initiating a long-acting reversible and permanent method relative to those who delivered per vagina. Studies have shown that women with HIV are more likely to opt for postpartum tubal ligation [36–38]. A study conducted among women who had been living with HIV for an average of 9.2 years showed that about half of them had been sterilised and expressed sterilisation regret [23]. However, a more recent study observed that the use of tubal ligation (3%) had reduced among women with HIV due to the increased availability of antiretroviral therapy (ART) [39]. Nonetheless, it is evident that caesarean section delivery is the primary facilitator of postpartum tubal ligation. Tubal ligation during caesarean section means that mothers do not have to present for another surgery. The choice of tubal ligation is also influenced by older age and having more than three children, which again established that these women do not want to have more children and used the opportunity of caesarean section to ensure their desire to limit childbirth is achieved. Doctors also find it convenient to perform bilateral tubal ligation during caesarean sections. Another plausible explanation is that clinicians often advise women with multiple deliveries via caesarean sections to choose a long-acting reversible or perform method.

Another important finding of this study is that parity is a significant determinant of choices of contraception in women with HIV. While women with three or fewer children are more likely to use short-acting contraception, women who have four or more children are significantly more likely to use irreversible contraception. This finding is consistent with previous studies [24–26]. Given that parity of four or more is far more than the fertility rate in South Africa [40], one could infer that the desire to limit childbearing motivated women’s decision to initiate a long-acting and permanent contraception.

### Study limitations

Given that there is a dearth of information on the immediate postpartum contraception among women in South Africa, this study specifically adds value to the existing knowledge on the topic. However, the limitations of the study cannot be ignored. The cross-sectional nature of the study means that any association reported cannot be interpreted as causation. Nonetheless, the large sample size ensures that the findings are reliable and representative of women with HIV in the Eastern Cape Province. The findings of this study further identify areas for further studies; such as the influence of prior contraception, providers’ bias and coercion, and informed counselling and choice on immediate postpartum contraception.

## Conclusion

We found a high prevalence of immediate postpartum contraception among parturient women living with HIV in our study setting, which is a strong indicator of the effectiveness of PMTCT and FP services’ integration. In addition, we observed a high prevalence of short-acting contraception (Nuristerat and Depo provera) among the parturient women living with HIV. Long-term follow up of this cohort to monitor retention in care and continuity of contraception including method switches will enhance our understanding of family planning needs of the study population. Innovative strategies to promote and enhance acceptance of long-acting contraception should be adopted to further mitigate the high rate of unplanned pregnancies in the region.

## Data Availability

All data will be made available by the corresponding author upon reasonable request.

## Abbreviations

FP: Family planning
LAPM: Long-acting permanent method
MTCT: Mother to child transmission of HIV
PMTCT: Prevention of mother to child transmission of HIV
WLWH: Women living with HIV
